# Apparent Longitudinal Variation in the Angiographic Expression of Myocardial Bridging: Reduced Dynamic Left Anterior Descending Artery Compression With Newly Detected Distal Left Circumflex Artery Bridging on Long-Term Follow-Up

**DOI:** 10.7759/cureus.108394

**Published:** 2026-05-06

**Authors:** Naresh Bohara, Adarsha Mahaseth, Jianwei Zhou, Asad Ullah, Xu Bing

**Affiliations:** 1 Interventional Cardiology, Yangzhou University Affiliated Northern Jiangsu People's Hospital, Yangzhou, CHN; 2 Internal Medicine, Nepalese Army Institute of Health Sciences, Kathmandu, NPL

**Keywords:** beta blocker therapy, coronary angiography, coronary artery disease, left anterior descending artery, left circumflex artery, myocardial bridging, serial imaging, systolic compression

## Abstract

Myocardial bridging (MB) is a congenital coronary anomaly characterized by an intramyocardial course of an epicardial coronary artery, resulting in dynamic systolic compression, most commonly involving the left anterior descending (LAD) artery. Although the anatomical substrate is generally considered congenital and structurally fixed, the angiographic expression of MB may vary according to hemodynamic conditions, pharmacologic therapy, imaging projection, and coexisting coronary artery disease. We report the case of a 71-year-old woman with hypertension and dyslipidemia who presented in 2020 with recurrent chest discomfort. Coronary angiography demonstrated a proximal-to-mid LAD MB with approximately 50-60% systolic compression and preserved flow, and she was managed conservatively with beta-blocker therapy. Six years later, she re-presented with recurrent chest pain. Repeat coronary angiography showed reduced dynamic systolic compression of the previously bridged LAD segment, which now appeared as an approximately 60% fixed luminal narrowing, along with newly detected dynamic MB in the distal branch of the left circumflex artery. These findings were interpreted as apparent longitudinal variation in angiographic expression rather than confirmed anatomical regression or de novo formation. The patient was managed medically without percutaneous coronary intervention because there was no flow-limiting stenosis, biomarkers were negative, and physiologic confirmation of ischemia was not obtained. This case highlights that serial coronary angiography in MB should be interpreted cautiously, particularly when imaging conditions and physiologic assessment are not standardized. For clinicians, the key implication is that apparent changes in MB over time may reflect functional modulation, imaging variability, and superimposed atherosclerosis rather than true structural evolution.

## Introduction

Myocardial bridging (MB) is a congenital coronary anomaly in which a segment of an epicardial coronary artery takes an intramyocardial or “tunneled” course beneath overlying myocardial fibers [1,2]. During systole, the bridged segment undergoes dynamic compression, producing the characteristic angiographic “milking effect,” with relative luminal re-expansion in diastole [1-3]. Although often considered a benign anatomical variant, MB may be associated with angina, myocardial ischemia, acute coronary syndromes, arrhythmias, and, in rare cases, sudden cardiac death [2-4].

The reported prevalence of MB varies substantially depending on the diagnostic modality employed. Autopsy and advanced imaging studies demonstrate considerably higher prevalence rates, often up to 40%-80%, whereas conventional coronary angiography detects functionally apparent bridging in a much smaller proportion of patients, typically ranging from 0.5% to 16% [1,3,5]. This discrepancy reflects both differences in detection sensitivity and the fact that many intramyocardial segments do not produce observable systolic compression on routine angiography [5]. Contemporary imaging modalities, including coronary computed tomography angiography, intravascular ultrasound, and Doppler-based techniques, have enhanced the anatomical and functional characterization of MB [3,6].

Anatomically, MB most frequently involves the left anterior descending (LAD) artery, accounting for approximately 67%-98% of cases in clinical and pathological series [3,7]. In contrast, involvement of the left circumflex (LCX) artery or right coronary artery (RCA) is uncommon, and LCX bridging is considered particularly rare [3,7-9]. Case reports describing LCX MB remain limited, underscoring its atypical nature [8-11]. Large coronary computed tomography angiography-based studies similarly confirm LAD predominance, with minimal or absent detection of LCX involvement in cohort analyses [10].

From a pathophysiological standpoint, MB influences coronary hemodynamics through mechanical systolic compression and delayed diastolic relaxation, which may reduce coronary flow reserve in susceptible individuals [2-4,7]. Additionally, altered shear stress proximal to the bridged segment has been implicated in the preferential development of atherosclerosis immediately proximal to the tunnel, while the intramyocardial segment itself is relatively spared [4,7]. The coexistence of MB and atherosclerotic coronary artery disease therefore represents a clinically important interaction that may modify symptom presentation and angiographic appearance [4].

Medical management remains the first-line therapeutic strategy for symptomatic MB. Beta-blockers are recommended because their negative chronotropic and inotropic effects reduce myocardial contractility and prolong diastole, thereby decreasing systolic compression and improving coronary perfusion [1,3,4]. Early angiographic studies demonstrated reduction of systolic narrowing following propranolol administration, providing foundational evidence for beta-blocker therapy in MB [12]. Invasive strategies, including surgical myotomy or intracoronary stenting, are generally reserved for refractory cases [3,4].

While MB is typically described as a congenital and anatomically stable variant [1,2], its angiographic visibility and apparent severity may vary according to heart rate, myocardial contractility, loading conditions, pharmacologic exposure, coronary projection, and coexisting atherosclerotic disease. Therefore, an apparent change in MB on serial angiography does not necessarily represent true anatomical regression, progression, or de novo formation. The specific knowledge gap addressed by this case is the limited description of how MB may appear differently on long-term follow-up coronary angiography, particularly when a previously dynamic LAD bridge appears less compressive while a distal LCX bridge is newly visualized. We present this case to highlight the distinction between true structural evolution and apparent longitudinal variation in angiographic expression, emphasizing the need for cautious interpretation of serial coronary imaging findings.

## Case presentation

A 71-year-old woman presented with recurrent episodes of chest discomfort. Her medical history was significant for long-standing hypertension of approximately 30 years’ duration, dyslipidemia, prior cholecystectomy, a remote history of cerebral infarction without residual neurological deficit, and chronic gastric ulcer disease. She had a prior history of coronary artery disease diagnosed in 2020, which had been managed conservatively without revascularization.

Initial presentation (2020)

In 2020, the patient presented with a three-year history of intermittent chest tightness and exertional dyspnea, with recent worsening in symptom frequency and intensity. The chest pain was pressure-like, non-radiating, and predominantly exertional, with partial relief at rest.

On examination, vital signs were stable, and cardiovascular examination was unremarkable without murmurs or signs of heart failure.

Electrocardiography demonstrated a sinus rhythm with nonspecific ST-T wave abnormalities. Transthoracic echocardiography showed preserved left ventricular systolic function with evidence of diastolic dysfunction. Chest computed tomography revealed multiple small pulmonary nodules, bilateral lower lobe emphysematous changes, and mild hepatic steatosis.

Laboratory investigations demonstrated dyslipidemia, with elevated triglycerides, total cholesterol, and low-density lipoprotein levels (Table 1).

**Table 1:** Baseline lipid profile at initial presentation (2020). LDL: Low-density lipoprotein cholesterol; mmol/L: millimoles per liter.

Parameter	Value	Units	Reference Range
Triglycerides	2.31	mmol/L	<1.7 mmol/L
Total Cholesterol	5.85	mmol/L	<5.2 mmol/L
LDL Cholesterol	3.59	mmol/L	<3.0 mmol/L

Coronary computed tomography angiography performed in March 2020 demonstrated a superficial myocardial bridge involving the proximal segment of the LAD artery.

**Figure 1:**
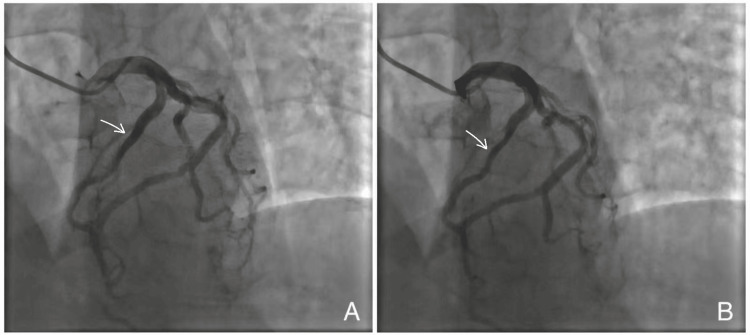
Coronary angiography (2020) demonstrating myocardial bridging in the left anterior descending artery. (A) Diastolic phase showing normal caliber of the proximal-to-mid segment of the left anterior descending artery (arrow). (B) Systolic phase demonstrating dynamic luminal narrowing of the same segment (arrow), producing the characteristic “milking effect,” consistent with functionally significant myocardial bridging.

**Figure 2:**
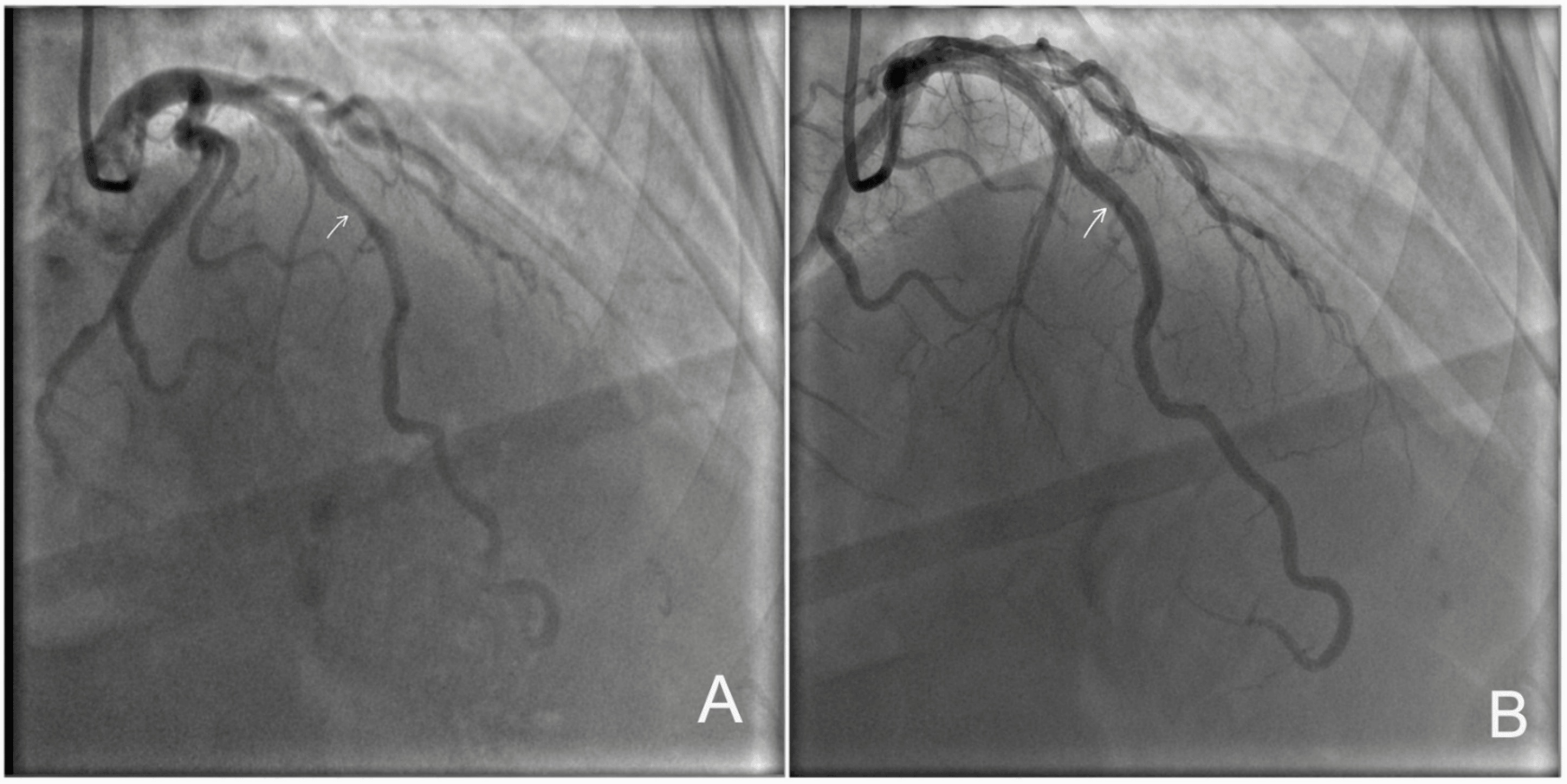
Detailed angiographic visualization of myocardial bridging in the left anterior descending artery (2020). (A) Systolic angiographic view demonstrating approximately 50–60% luminal compression of the proximal-to-mid left anterior descending artery bridged segment (arrow), consistent with the characteristic “milking effect.” (B) Corresponding diastolic angiographic view showing re-expansion of the same left anterior descending artery segment (arrow), confirming the dynamic nature of myocardial bridging.

Subsequent invasive coronary angiography confirmed MB of the proximal-to-mid LAD. During diastole, the LAD demonstrated normal vessel caliber, whereas systolic imaging revealed dynamic luminal narrowing of the same segment, producing the characteristic “milking effect” (Figures 1A, 1B). Detailed angiographic visualization demonstrated approximately 50-60% systolic compression with complete diastolic re-expansion, confirming the dynamic nature of MB (Figures 2A, 2B).

**Figure 3:**
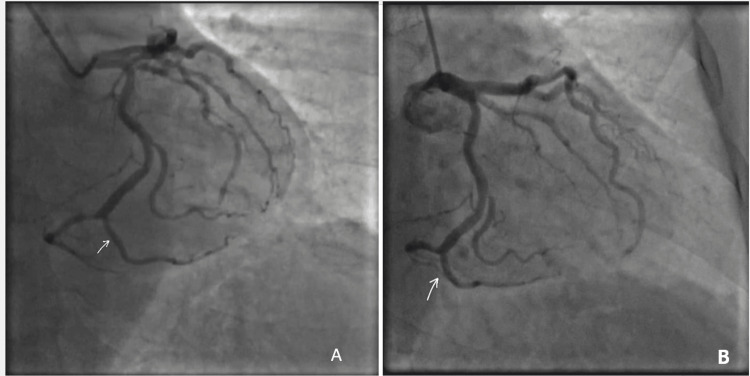
Coronary angiographic images of the distal left circumflex artery (2020). (A) Systolic angiographic view of the distal left circumflex artery/terminal branch showing the arrowed segment without convincing focal systolic compression or a definite “milking effect.” (B) Corresponding diastolic view of the same distal left circumflex artery/terminal branch (arrow) without clear evidence of myocardial bridging.

Evaluation of the LCX artery at that time did not demonstrate MB. Both systolic and diastolic views of the distal LCX/terminal branch showed no convincing focal compression or “milking effect,” indicating the absence of functional bridging (Figures 3A, 3B).

The right coronary artery demonstrated moderate atherosclerotic changes with preserved distal flow. Intravascular ultrasound of the right coronary artery lesion revealed a minimum lumen area of 5.03 mm², suggesting no hemodynamically significant stenosis.

During the interval between 2020 and 2026, the patient remained on conservative medical therapy, including beta-blocker-based antianginal treatment and lipid-lowering therapy. No coronary revascularization was performed during this period. There was no interim myocardial infarction, heart failure hospitalization, or repeat invasive coronary evaluation before the 2026 presentation.

Follow-up presentation (2026)

Six years later, the patient presented again with intermittent chest pain and chest tightness of one-month duration. The symptoms were similar in nature to prior episodes but had increased in frequency. She also reported associated fatigue and mild exertional dyspnea.

On examination, blood pressure was 136/84 mmHg and heart rate was 80 beats per minute. Cardiovascular examination remained unremarkable.

**Figure 4:**
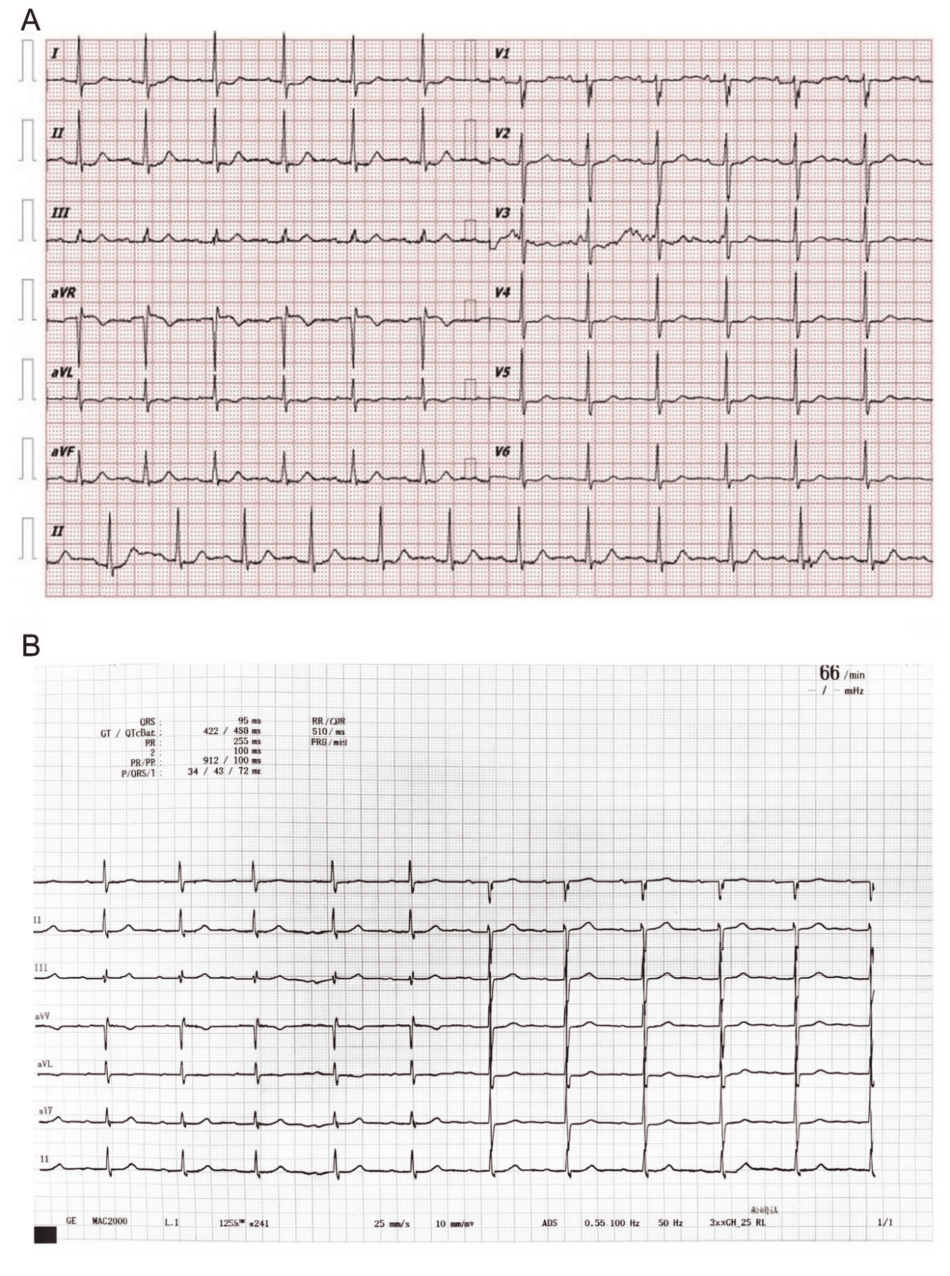
Electrocardiographic comparison between 2020 and 2026. (A) Twelve-lead electrocardiogram obtained at the initial presentation in 2020 demonstrating sinus rhythm with nonspecific ST–T wave abnormalities. (B) Follow-up twelve-lead electrocardiogram obtained in 2026 demonstrating sinus rhythm with persistent nonspecific ST–T wave abnormalities, without new acute ischemic changes compared with the prior tracing.

Electrocardiography again demonstrated sinus rhythm with persistent nonspecific ST-T abnormalities, without new ischemic changes compared to prior recordings (Figure 4). Transthoracic echocardiography revealed preserved left ventricular systolic function (ejection fraction 56%) without regional wall motion abnormalities.

Comprehensive laboratory evaluation revealed multiple abnormalities, including anemia, leukocytosis, renal dysfunction, hyperglycemia, and dyslipidemia (Table 2).

**Table 2:** Laboratory investigations at re-presentation (2026). eGFR: Estimated glomerular filtration rate; HbA1c: Glycated hemoglobin; HDL: High-density lipoprotein cholesterol; LDL: Low-density lipoprotein cholesterol; NT-proBNP: N-terminal pro–B-type natriuretic peptide; g/L: grams per liter; ×10⁹/L: ×10⁹ cells per liter; mmol/L: millimoles per liter; µmol/L: micromoles per liter; mL/min/1.73 m²: milliliters per minute per 1.73 square meters of body surface area; ng/mL: nanograms per milliliter; pg/mL: picograms per milliliter; %: percentage.

Parameter	Value	Units	Reference Range
Hemoglobin	113 ↓	g/L	120–160 g/L
White Blood Cells	10.08 ↑	×10⁹/L	4–10 ×10⁹/L
Platelets	235	×10⁹/L	150–400 ×10⁹/L
Urea	12.07 ↑	mmol/L	2.5–7.1 mmol/L
Creatinine	118 ↑	µmol/L	44–106 µmol/L
eGFR	40.17 ↓	mL/min/1.73 m²	>90
HbA1c	7.3 ↑	%	<5.7%
Triglycerides	3.25 ↑	mmol/L	<1.7 mmol/L
Total Cholesterol	4.62	mmol/L	<5.2 mmol/L
HDL Cholesterol	1.08 ↓	mmol/L	>1.2 mmol/L
LDL Cholesterol	1.86	mmol/L	<3.0 mmol/L
Troponin I	<0.012	ng/mL	<0.04 ng/mL
NT-proBNP	66.5	pg/mL	<125 pg/mL

These findings indicated progression of the patient’s cardiometabolic risk profile compared with the initial presentation, particularly with newly recognized diabetes mellitus, renal dysfunction consistent with chronic kidney disease, persistent hypertriglyceridemia, and low high-density lipoprotein cholesterol. These abnormalities were clinically relevant because they may have contributed to progression of atherosclerotic coronary artery disease and to the fixed stenotic appearance observed in the LAD on follow-up angiography.

**Figure 5:**
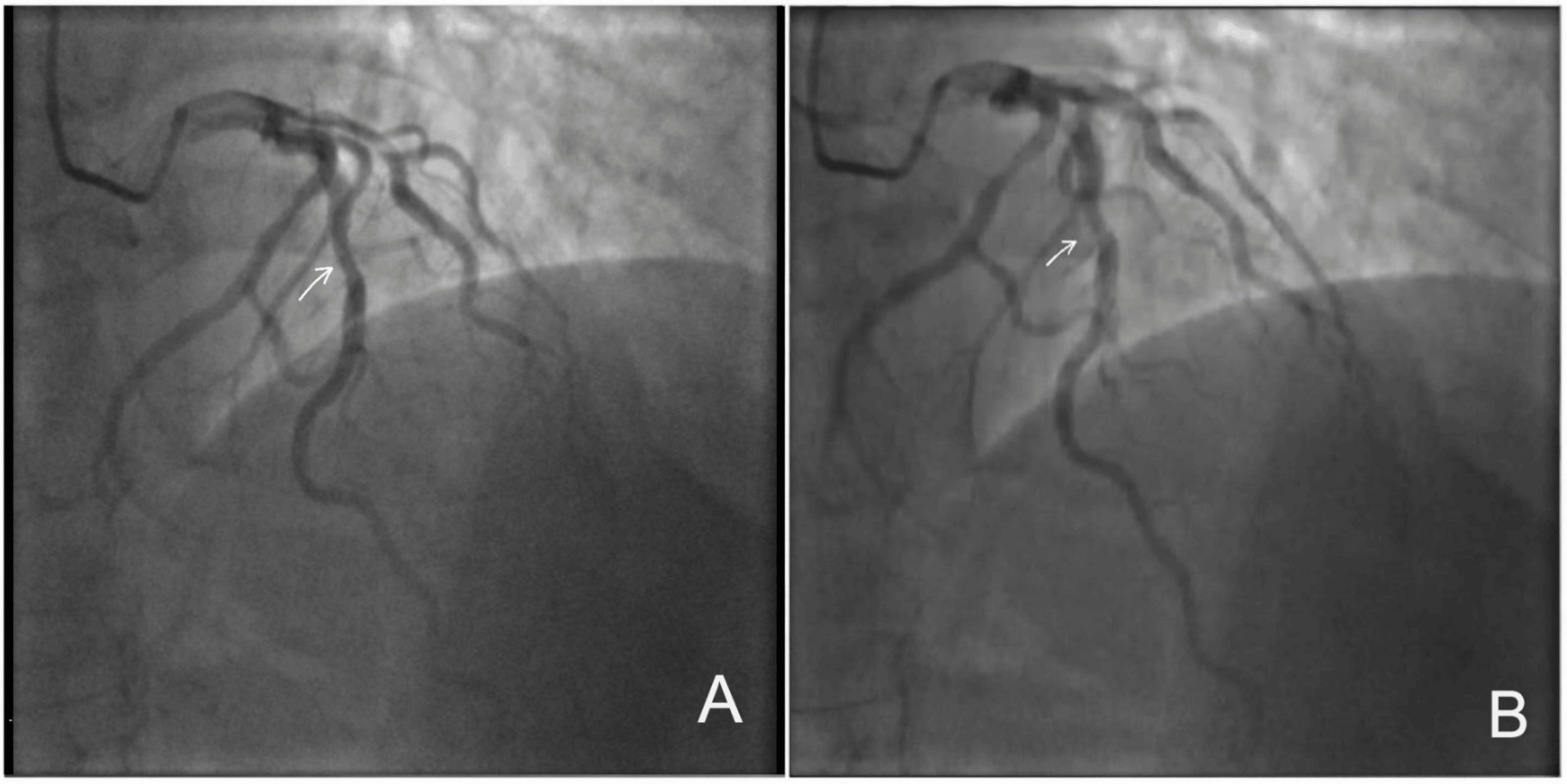
Coronary angiography (2026) of the left anterior descending artery demonstrating altered lesion morphology. (A) Angiographic view showing an approximately 60% fixed luminal narrowing in the proximal-to-mid left anterior descending artery at the arrowed segment. (B) Corresponding view of the same arrowed segment without clear demonstrable dynamic systolic compression or a definite “milking effect,” consistent with altered angiographic appearance of a previously bridged segment and possible superimposed atherosclerotic change.

Repeat coronary angiography performed in January 2026 demonstrated significant changes compared to the prior study. The previously bridged proximal-to-mid LAD segment now appeared as an approximately 60% fixed luminal narrowing. Notably, corresponding angiographic views did not demonstrate clear dynamic systolic compression or a definite “milking effect,” suggesting an altered angiographic appearance of a previously bridged segment, potentially reflecting pharmacologic modulation and/or superimposed atherosclerotic change (Figures 5A, 5B).

**Figure 6:**
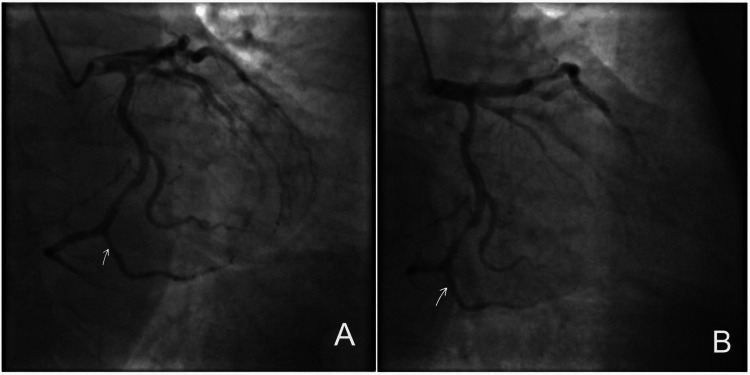
Coronary angiographic visualization of myocardial bridging in the distal left circumflex artery (2026). (A) Systolic angiographic view demonstrating focal compression/narrowing of the arrowed distal left circumflex artery/terminal branch, producing a “milking effect.” (B) Corresponding diastolic angiographic view showing re-expansion of the same arrowed segment with preserved distal opacification, consistent with myocardial bridging.

In contrast, a myocardial bridge was identified in the distal LCX/terminal branch. Systolic angiographic images demonstrated focal compression of the vessel segment with a characteristic “milking effect,” while diastolic imaging showed re-expansion of the same segment with preserved distal opacification, confirming dynamic MB (Figures 6A, 6B).

The right coronary artery demonstrated diffuse atherosclerotic plaque without flow-limiting stenosis. The 2026 coronary angiogram was performed using standard coronary angiographic projections comparable to those used during the 2020 evaluation, with systolic and diastolic frames reviewed for the LAD and LCX territories. Intracoronary nitroglycerin was not administered before acquisition of the images used for comparison. The resting heart rate at the 2026 presentation was 80 beats per minute, lower than the approximately 96 beats per minute documented during the 2020 presentation. Although the same coronary territories were assessed using comparable standard views, exact frame-by-frame standardization was limited by differences in projection angle, catheter position, loading conditions, and beat-to-beat heart rate during image acquisition. Accordingly, the findings should be interpreted as apparent longitudinal variation in angiographic expression rather than confirmed anatomical evolution of MB.

Overall, serial evaluation demonstrated apparent longitudinal variation in the angiographic expression of MB, with reduced visible dynamic compression of the previously bridged LAD segment, a fixed stenotic appearance in the same region, newly visualized dynamic compression in the distal LCX/terminal branch, and progression of cardiometabolic risk factors, including diabetes mellitus and chronic kidney disease.

Management

Based on the clinical, laboratory, and angiographic findings, the patient was diagnosed with coronary artery disease, unstable angina, MB of the distal LCX artery, hypertension, type 2 diabetes mellitus, and chronic kidney disease.

Percutaneous coronary intervention was not performed because the LAD lesion was visually estimated at approximately 60% without angiographic evidence of critical flow limitation, the right coronary artery showed diffuse plaque without flow-limiting stenosis, cardiac biomarkers were negative, and echocardiography did not demonstrate regional wall motion abnormality. In addition, the distal LCX finding was consistent with dynamic MB rather than a fixed obstructive lesion suitable for stenting. Physiologic assessment with fractional flow reserve (FFR), instantaneous wave-free ratio (iFR), or diastolic FFR was considered clinically relevant but was not performed; therefore, the functional significance of the LAD narrowing and LCX bridging could not be objectively confirmed. In this context, the treating team favored optimized medical therapy rather than revascularization.

She was managed medically with antiplatelet therapy, statin therapy, beta-blocker-based antianginal treatment, antihypertensive therapy, dapagliflozin for diabetes and cardiometabolic risk reduction, and proton pump inhibitor therapy. Identification of distal LCX MB did not lead to an invasive intervention but reinforced the decision to continue beta-blocker-based medical therapy and risk-factor optimization, with clinical follow-up recommended for recurrent or refractory symptoms.

## Discussion

MB is classically defined as an epicardial coronary artery segment that courses intramyocardially and undergoes systolic compression, producing the characteristic angiographic “milking effect” [1]. The clinical relevance of MB varies widely, ranging from incidental findings to exertional angina, acute coronary syndromes, arrhythmias, and syncope [2,3]. In the present case, coronary angiography in 2020 demonstrated a proximal-to-mid LAD artery MB with approximately 50%-60% systolic compression and preserved TIMI 3 flow, representing a typical angiographic phenotype of symptomatic MB described in prior literature [1,2]. The patient’s recurrent chest discomfort is also consistent with the recognized clinical spectrum of symptomatic bridging [2,3,13].

Consistent with established observations, the LAD was the involved vessel at baseline, reflecting the well-documented predominance of this artery in MB [3]. In contrast, involvement of the LCX or right coronary artery is relatively uncommon, and the identification of dynamic compression in the distal LCX/terminal branch on repeat angiography therefore represents an unusual finding. However, because MB is widely regarded as a congenital or structural anatomical variant rather than an acquired lesion [1,2], the LCX finding should not be interpreted as definite de novo formation. Major reviews do not describe routine migration or new development of bridging sites over time [1,3]. Accordingly, the present finding is best interpreted as newly visualized or newly detected LCX MB on serial angiography, potentially related to differences in angiographic projection, hemodynamic conditions, pharmacologic exposure, or detection sensitivity.

A key feature of this case is the apparent change in the angiographic expression of the LAD artery lesion over time. In 2020, dynamic systolic compression was clearly demonstrated, whereas in 2026 the same segment appeared as an approximately 60% fixed luminal stenosis without a demonstrable “milking effect.” Several mechanisms described in the literature may account for this observation. First, beta-blockers are considered first-line therapy for symptomatic MB because their negative chronotropic and inotropic effects reduce myocardial contractility and prolong diastole, thereby diminishing systolic compression [1,3,4]. Early angiographic evidence demonstrated reduction in systolic narrowing following propranolol administration, establishing that the severity of compression can be pharmacologically modulated [12]. In this patient, long-term metoprolol therapy was associated with a reduction in resting heart rate from 96 beats per minute in 2020 to 80 beats per minute in 2026, supporting this physiological mechanism. The absence of clearly visible dynamic compression on follow-up imaging may therefore represent reduced angiographic expression of functional systolic compression rather than disappearance of the anatomical bridge [12].

Second, angiographic manifestations of MB are known to be highly sensitive to hemodynamic and pharmacologic conditions. It has been demonstrated that nitroglycerin can accentuate systolic narrowing in patients with angiographic bridging, highlighting that the degree of dynamic compression may vary depending on physiological state at the time of imaging [14]. This variability provides a plausible explanation for the prominence of dynamic compression in 2020 and its apparent absence in 2026.

Third, the relationship between MB and atherosclerosis provides an additional interpretative framework. Several studies have shown that the intramyocardial segment of a bridged artery is relatively spared from atherosclerosis, whereas the segment proximal to the bridge is more prone to plaque formation due to altered shear stress [15,16]. The coexistence of MB and atherosclerotic coronary artery disease has been well described on imaging studies [17]. In the present case, the transition from dynamic systolic compression to a fixed stenotic appearance in the LAD artery may reflect progressive atherosclerosis in the proximal or adjacent segment of a previously bridged region. Given the patient’s advanced age and accumulation of cardiometabolic risk factors, including hypertension, newly diagnosed diabetes mellitus, and chronic kidney disease, progressive atherosclerotic disease is biologically plausible. Importantly, this does not imply resolution of the myocardial bridge but rather a shift in the dominant angiographic phenotype from dynamic compression to fixed luminal narrowing.

The coexistence of MB and atherosclerotic coronary artery disease is clinically significant, as symptomatic presentations may result from a complex interplay between dynamic systolic compression and fixed obstructive lesions [13,17]. It has been emphasized that ischemia in MB may involve not only systolic narrowing but also delayed diastolic relaxation and altered coronary flow dynamics [13]. In this patient, recurrent anginal symptoms are likely multifactorial, reflecting the combined effects of MB, fixed stenosis, and diffuse atherosclerosis.

Taken together, this case is consistent with established literature regarding the definition, anatomical distribution, clinical presentation, and pharmacologic modulation of MB [1-3,12]. However, the serial observation of reduced visible dynamic compression in the previously bridged LAD segment, together with newly visualized dynamic compression in the distal LCX/terminal branch, represents an unusual longitudinal angiographic finding. Because MB is generally considered congenital, this phenomenon should be interpreted cautiously as variation in angiographic expression, functional modulation, and detection over time rather than definitive anatomical regression, migration, or de novo formation. These findings highlight the dynamic interplay between coronary anatomy, hemodynamics, pharmacotherapy, and progressive atherosclerotic disease, underscoring the importance of careful interpretation of serial coronary imaging.

From a practical management perspective, this case emphasizes that apparent changes in MB on serial angiography should not automatically prompt revascularization unless there is objective evidence of functionally significant ischemia or refractory symptoms despite optimized medical therapy. In patients with intermediate fixed stenosis coexisting with MB, physiologic assessment such as FFR, iFR, or preferably diastolic physiologic indices may help determine whether symptoms are driven by fixed atherosclerotic obstruction, dynamic bridging, or both. When physiologic testing is not performed, management should be individualized and should prioritize antianginal therapy, heart-rate control, and aggressive modification of atherosclerotic risk factors, with further testing or intervention reserved for persistent or worsening symptoms.

Limitations

This case report has several important limitations that should be acknowledged when interpreting the findings. First, physiologic assessment was not performed. FFR, iFR, or diastolic FFR were not obtained during either the initial or follow-up angiographic evaluations. As a result, the functional significance of the previously observed LAD MB, the intermediate fixed LAD stenosis seen on follow-up, and the newly visualized distal LCX MB could not be objectively quantified. This limits the ability to determine whether the patient’s recurrent symptoms were primarily related to fixed atherosclerotic stenosis, dynamic MB, microvascular dysfunction, or a combination of these mechanisms.

Second, intravascular imaging of the LAD was not performed at follow-up. Although intravascular ultrasound (IVUS) was used to assess the RCA lesion in 2020, repeat IVUS or optical coherence tomography of the LAD in 2026 could have provided additional anatomical detail. Such imaging may have helped differentiate progressive atherosclerotic plaque formation from altered angiographic visualization of a previously bridged segment.

Third, the serial angiographic comparison was limited by the absence of a fully standardized imaging protocol. Although standard coronary angiographic projections were used and comparable LAD and LCX territories were reviewed, exact frame-by-frame standardization between the 2020 and 2026 studies could not be confirmed. Differences in projection angle, catheter position, loading conditions, and beat-to-beat heart rate during image acquisition may have affected the apparent severity of systolic compression. Intracoronary nitroglycerin was not administered before acquisition of the 2026 images used for comparison; however, nitrate use during the 2020 angiographic acquisition was not fully documented. These factors limit direct comparison between the two studies.

Fourth, the apparent reduction in visible LAD systolic compression should not be interpreted as anatomical resolution of MB. MB is generally considered a congenital structural anomaly, and its angiographic expression may be influenced by heart rate, myocardial contractility, pharmacologic therapy, and hemodynamic conditions. In this patient, the lower resting heart rate in 2026 compared with 2020 and long-term beta-blocker therapy may have contributed to reduced visible dynamic compression on follow-up angiography.

Fifth, the newly visualized distal LCX MB cannot be definitively classified as a new anatomical development. Given the congenital nature of MB, this finding is more plausibly explained by delayed recognition, differences in angiographic projection, variable functional expression, or detection sensitivity rather than true de novo formation.

Sixth, non-invasive ischemia testing, such as stress echocardiography, myocardial perfusion imaging, or cardiac magnetic resonance imaging, was not performed during follow-up. Therefore, the relationship between the angiographic findings and the patient’s recurrent symptoms remains inferential rather than directly demonstrated.

Finally, this is a single-patient observational report, and the findings are inherently limited in generalizability. The apparent longitudinal variation observed in this case may not represent typical MB behavior. Therefore, the case should be interpreted as an example of angiographic variability and functional modulation over time rather than evidence of true anatomical evolution.

## Conclusions

This case illustrates apparent longitudinal variation in the angiographic expression of MB over six years. The previously documented LAD bridged segment showed reduced visible dynamic systolic compression on follow-up angiography and appeared as an intermediate fixed stenotic lesion, while dynamic compression was newly visualized in the distal LCX/terminal branch. Because MB is generally considered a congenital structural anomaly, these findings most likely reflect functional modulation, imaging variability, pharmacologic influence, and coexisting atherosclerotic disease rather than true anatomical regression, migration, or de novo formation. Clinicians should interpret serial angiographic changes in MB cautiously and consider physiologic assessment when management decisions depend on the functional significance of intermediate stenosis or dynamic compression.
